# PROMOTING PURPOSEFUL RETIREMENT IN NORTH DAKOTA AND SOUTH DAKOTA

**DOI:** 10.1093/geroni/igae098.3506

**Published:** 2024-12-31

**Authors:** Leacey Brown, Jane Strommen

**Affiliations:** South Dakota State University Extension, Rapid City, South Dakota, United States; North Dakota State University Extension, Fargo, North Dakota, United States

## Abstract

A sense of purpose is correlated with a variety of outcomes in older age. Outcomes associated with purpose include mortality, longevity, psychological health, dementia/memory, physical health, and health promotion. Yet, many adults may not view older age and retirement as a time to live with purpose. As a result, educational programs are needed to help adults learn how they might incorporate purpose into their retirement planning. As the land-grant universities in their respective states, North Dakota State University and South Dakota State University are well positioned to fill this void. Both organizations are tasked with providing education and outreach on a variety of topics for individuals and families. For this reason, they came together to develop the Purposeful Retirement Book Club. This program was first piloted in 2020 and has been offered five times, reaching 81 adults. This poster will describe the value of including purpose in retirement planning, discuss evaluation results and lessons learn piloting the program, as well as strategies other organizations might use to implement this program in their community. Participants will also learn about the facilitator guide that was developed following the pilot of the program. This guide is available for anyone interested in offering the program.

